# Genetic diversity and natural selection of *Plasmodium knowlesi* merozoite surface protein 1 paralog gene in Malaysia

**DOI:** 10.1186/s12936-018-2256-y

**Published:** 2018-03-14

**Authors:** Md Atique Ahmed, Muh Fauzi, Eun-Taek Han

**Affiliations:** Department of Medical Environmental Biology and Tropical Medicine, School of Medicine, Kangwon National University, Chuncheon, Gangwon-do 24341 Republic of Korea

**Keywords:** *Plasmodium knowlesi*, Merozoite surface protein 1 paralog, Genetic diversity, Natural selection, Sub-populations, Haplotypes

## Abstract

**Background:**

Human infections due to the monkey malaria parasite *Plasmodium knowlesi* is on the rise in most Southeast Asian countries specifically Malaysia. The C-terminal 19 kDa domain of PvMSP1P is a potential vaccine candidate, however, no study has been conducted in the orthologous gene of *P. knowlesi.* This study investigates level of polymorphisms, haplotypes and natural selection of full-length *pkmsp1p* in clinical samples from Malaysia.

**Methods:**

A total of 36 full-length *pkmsp1p* sequences along with the reference H-strain and 40 C-terminal *pkmsp1p* sequences from clinical isolates of Malaysia were downloaded from published genomes. Genetic diversity, polymorphism, haplotype and natural selection were determined using DnaSP 5.10 and MEGA 5.0 software. Genealogical relationships were determined using haplotype network tree in NETWORK software v5.0. Population genetic differentiation index (*F*_*ST*_) and population structure of parasite was determined using Arlequin v3.5 and STRUCTURE v2.3.4 software.

**Results:**

Comparison of 36 full-length *pkmsp1p* sequences along with the H-strain identified 339 SNPs (175 non-synonymous and 164 synonymous substitutions). The nucleotide diversity across the full-length gene was low compared to its ortholog *pvmsp1p.* The nucleotide diversity was higher toward the N-terminal domains (*pkmsp1p*-*83* and *30*) compared to the C-terminal domains (*pkmsp1p*-*38, 33* and *19*). Phylogenetic analysis of full-length genes identified 2 distinct clusters of *P. knowlesi* from Malaysian Borneo. The 40 *pkmsp1p*-*19* sequences showed low polymorphisms with 16 polymorphisms leading to 18 haplotypes. In total there were 10 synonymous and 6 non-synonymous substitutions and 12 cysteine residues were intact within the two EGF domains. Evidence of strong purifying selection was observed within the full-length sequences as well in all the domains. Shared haplotypes of 40 *pkmsp1p*-*19* were identified within Malaysian Borneo haplotypes.

**Conclusions:**

This study is the first to report on the genetic diversity and natural selection of *pkmsp1p*. A low level of genetic diversity and strong evidence of negative selection was detected and observed in all the domains of *pkmsp1p* of *P. knowlesi* indicating functional constrains. Shared haplotypes were identified within *pkmsp1p*-*19* highlighting further evaluation using larger number of clinical samples from Malaysia.

**Electronic supplementary material:**

The online version of this article (10.1186/s12936-018-2256-y) contains supplementary material, which is available to authorized users.

## Background

Malaria is a major health threat in many parts of the globe and causes high mortality and morbidity, with 212 million cases of malaria occurring globally in 2015, with 429,000 deaths [1]. *Plasmodium knowlesi*, a parasite of long- and pig-tailed macaques is now considered as the fifth *Plasmodium* species infecting humans and is an emerging threat in most Southeast Asian countries [2–6]. Human infections due to *P. knowlesi* are increasingly reported from a number of the Southeast Asian countries, including Malaysia [4, 7, 8], Singapore [9], Myanmar [10], Vietnam [11], Indonesia [12], Philippines [13], Cambodia [14], India [15] and Thailand [16]. In Malaysia, the public health threat posed by the zoonotic malaria parasite *P. knowlesi* appears to be growing, with increasing number of human infections being reported from Peninsular Malaysia as well as Malaysian Borneo [4, 8, 17], which highlights the need of effective measures for control as well as development of effective vaccines.

The parasite is the only human and non-human primate malaria that has a 24-h erythrocytic cycle; rapid increase in parasitaemia has been shown to be associated with the development of severe malaria in humans and is a common cause for severe and fatal malaria in Malaysian Borneo [3, 18–20]. Approximately 70% of malaria cases reported from the Kapit division of Sarawak [18] and 78% from Kudat, Sabah are due to *P. knowlesi* [8]. Recent *P. knowlesi* genomic and microsatellite-based studies from Sarawak, Malaysian Borneo have shown that there are at least 3 sub-populations of the parasite and 2 of the populations were associated with long-tailed (*Macaca fascicularis)* and pig-tailed (*Macaca nemestrina*) macaques from Malaysian Borneo [21–23]. Analysis of mitochondrial genes in *P. knowlesi* isolates from patients and macaques also identified two distinct clusters which clustered geographically to Malaysian mainland and Malaysian Borneo [24]. These studies have highlighted the complexity of *P. knowlesi* infections in humans and challenges for control as well as vaccine design.

Malaria vaccine development is hindered by natural polymorphisms within blood-stage candidate antigens and, therefore, it is critical to determine the pattern of diversity, natural selection and population structure in vaccine candidates and its significance for the acquisition and effectiveness of protective immunity. For example, the protection conferred by the most advanced candidate subunit vaccine is RTS,S/AS01, which targets the *Plasmodium falciparum* circumsporozoite protein (PfCSP) was found to be rapidly declining in sub-Saharan Africa [25]. A recent study showed that polymorphisms within the merozoite invasion genes (normocyte binding protein xa and xb*, nbpxa* and *nbpxb*) of *P. knowlesi* were linked to hyperparasitaemia and disease severity in human infections [26]. Potential vaccine candidates like Duffy binding protein (DBP), merozoite surface protein (MSP) 1 and 3, normocyte binding protein xa have recently been studied from *P. knowlesi* clinical isolates [27–29].

*Plasmodium knowlesi* is phylogenetically closely related to *Plasmodium vivax* [30]. In *P. vivax,* MSP1 is a well-known blood stage antigen which localizes on the merozoite surface and the C-terminal 19 kDa domain is responsible for binding to erythrocytes and antibodies against the C-terminal fragment of MSP1 shows parasite invasion inhibitory properties [31, 32]. Most of the merozoite surface proteins (e.g., MSP1, MSP2, MSP4, MSP5, MSP8, and MSP10) contain 1 or 2 copies of a conserved epidermal growth factor (EGF)-like domain at the carboxyl terminal that are anchored to the membrane via glycosylphosphatidylinositol (GPI) membrane anchor [33, 34].

Recently, a novel vaccine candidate *P. vivax* merozoite surface protein 1 paralog (PvMSP1P-19; PlasmoDB accession no. PVX_099975) a glycosylphosphatidylinositol (GPI)-anchored protein which is expressed on the merozoite surface during blood-stage development and binds to erythrocytes is reported [35]. Studies have also reported the PvMSP1-19 to be immunogenic and antigenicity has been reported in *P. vivax*-infected patients [36, 37]. The primary structure of PvMSP1P is similar to PvMSP1 and they contain a putative GPI-anchored motif and 2 epidermal growth factor (EGF)-like domains at the C terminus [38]. The predicted molecular mass is about 215 kDa, which is similar to that of PvMSP1. These studies have also proved that the protein undergoes proteolytic processing during the invasion process similar to PvMSP1 to produce 83, 30, 38, 33 and 19 kDa domains [35]. The C-terminal 19 kDa domain which contains the EGF-like domains are mostly conserved among all the MSPs studied to date and thus are speculated to possess conserved binding activity to host erythrocytes. Studies on genetic polymorphism of PvMSP1P C-terminal domain in worldwide isolates indicated low levels of polymorphisms and thus might serve as a potential vaccine candidate [39]. However, no study has been done to characterize the PkMSP1P which is an ortholog gene.

In this study firstly, the domains of PkMSP1P protein were characterized based on the amino acid sequence alignment to its ortholog PvMSP1P sequence. Then the level of sequence diversity, natural selection using full-length genes at each of the domains (83, 30, 38, 33 and 19 kDa domains) were determined using 34 clinical isolates and 2 laboratory lines (along with the H-strain) of Malaysia. Based on the 19 kDa domain (PkMSP1P-19), the shared haplotypes were identified within 40 isolates and the population structure was determined based on the 42 kDa domain (PkMSP1P-42) from clinical isolates from 4 geographically different regions in Malaysia. The information obtained from this study will be helpful for future rational design and formulation of a vaccine against *P. knowlesi,* and will aid in the understanding of transmission dynamics of *P. knowlesi* within Malaysia.

## Methods

### PkMSP1P sequence data

PkMSP1P sequences were downloaded for 36 clinical isolates originating from Kapit, Betong and Sarikei in Malaysian Borneo, 4 long-time isolated lines from Peninsular Malaysia along with the H-strain (PKNH_0728800) (Additional file 1) [23]. The sequence data accession numbers are listed in (Additional file 2). Of these, 36 sequences were used for characterization of the full-length PkMSP1P gene (Additional file 2). Signal peptide for the full-length PkMSP1P domain was predicted using Signal IP 3.0 and Phobious prediction software [40, 41]. The PkMSP1P domains were characterized based on the published ortholog of PvMSP1P (PVX_099975) [35]. Phylogenetic analysis was conducted using deduced amino acid sequences from 7 PkMSP1P full-length from Malaysian Borneo, 2 laboratory lines from Peninsular Malaysia; reference H-strain (PKNH_0728800) and the Malayan Strain (Pk1A PKNOH_S06430900) along with other ortholog members of *P. vivax* Sal-1 (PVX_099975), *P. vivax* P01 (PVP010728800), *Plasmodium cynomolgi* (PcYB073760) and *Plasmodium ovale curtisi* (PoCGH0107037800) using unrooted neighbour-joining (NJ) method also described in MEGA5. Bootstrap replicates of 1000 were used to test the robustness of the trees.

### Sequence diversity and natural selection

Sequence diversity (π), defined as the average number of nucleotide differences per site between two sequences within the sequences, was determined by DnaSP v5.10 software [42]. Number of polymorphic sites, number of synonymous and non-synonymous substitutions, haplotype diversity (Hd), number of haplotypes (h) within the *pkmsp1p* sequences were also determined by DnaSP software.

To investigate departure from neutrality, Tajima’s D analysis was conducted [43]. Under neutrality, Tajimas D is expected to be 0. Significantly, positive Tajima’s D values indicate recent population bottleneck or balancing selection, whereas negative values suggest population expansion or negative selection. The rates of synonymous (dS) and non-synonymous (dN) mutations were estimated and compared by the Z-test (*P* < 0.05) in MEGA5 using the Nei and Gojobori’s method with the Jukes and Cantor (JC) correction and 1000 bootstrap replications [44].

### Haplotype network

Haplotype diversity (Hd) and number of haplotypes (H) were determined using DnaSP software. Genealogical relationships between the *pkmsp1p*-*19* haplotypes were constructed using the median-joining method in NETWORK software (version 4.6.1.2, Fluxus Technology Ltd, Suffolk, UK).

### Population genetic structure analysis

To define genetic structure of the *P. knowlesi* parasite population in Malaysia based on the *msp1p*, STRUCTURE software (version 2.3.4) was used that deploys the Bayesian model based clustering approach. The most probable number of populations (K) was determined using an admixture model. Since the 19 kDa domain is largely conserved in all *Plasmodium* species, the 42 kDa domain (19 and 33 kDa) was used for population structure analysis. All sample data were run for values K = 1–6, each with a total of 15 iterations, 100,000 Markov Chain Monte Carlo (MCMC) generations for each run after a burn-in of 50,000 steps. The most likely number K in the data was estimated by calculating ΔK values and identifying the K value that maximizes the log probability of data, lnP(D). The most probable K value was then calculated according to Evanno’s method by using the webpage interface STRUCTURE Harvester. The ARLEQUIN software (version 3.5.1.3, University of Berne, Berne, Switzerland) was used to compute pairwise differences (*F*_*ST*_) between populations (i.e., Sarikei, Betong, Kapit and Peninsular Malaysia) with 10,100 permutations. *F*_*ST*_ is a comparison of the sum of genetic variability within and between populations on the basis of the differences in allelic frequencies. *F*_*ST*_ values are interpreted as no (0), low (> 0–0.05), moderate (0.05–0.15), and high (0.15–0.25) genetic differentiation.

## Results

### Genetic diversity and natural selection of full-length *pkmsp1p*

**Fig. 1:**
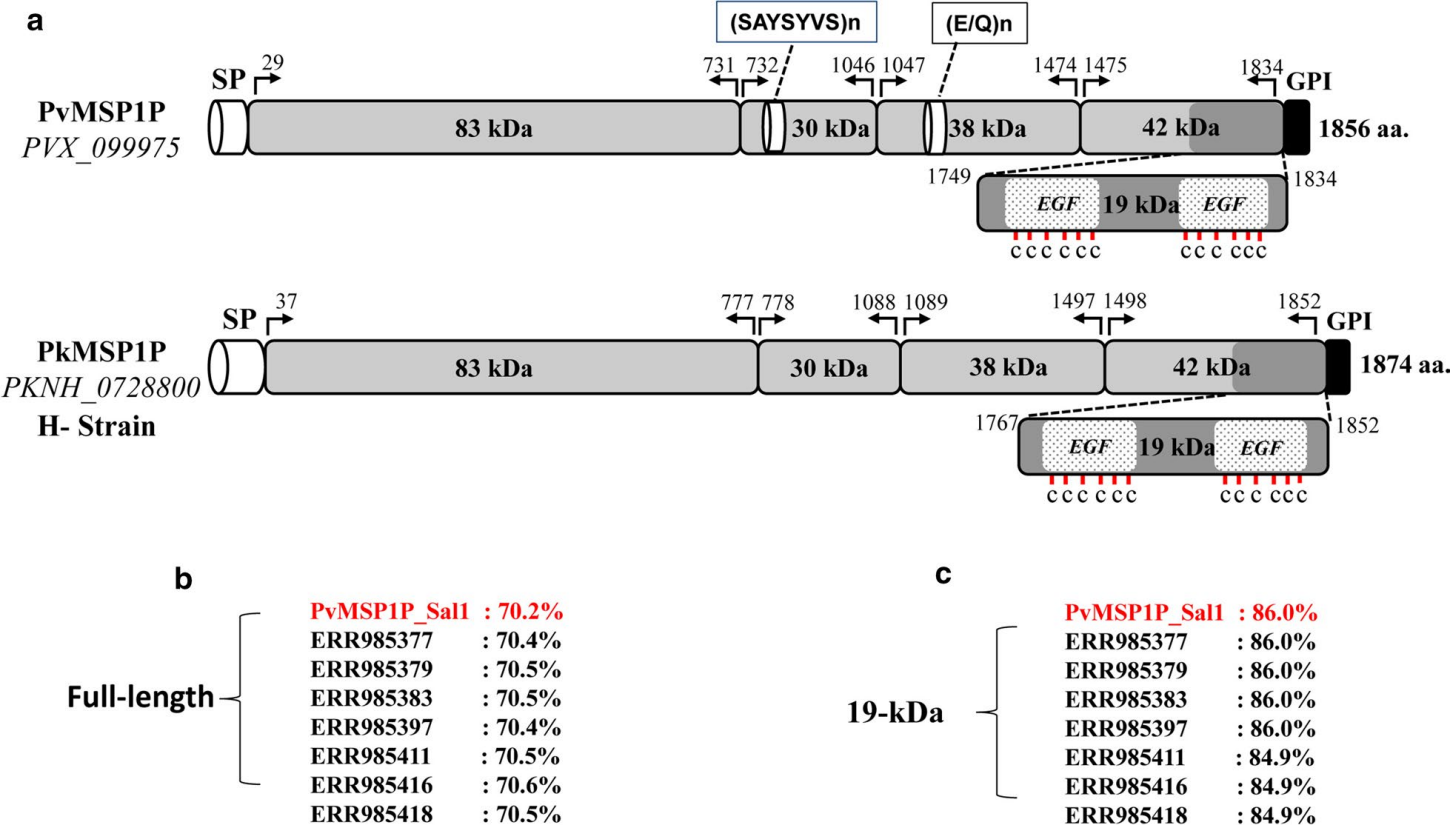
**a** Schematic diagram of *Plasmodium vivax* (PVX_099975) and *Plasmodium knowlesi* merozoite surface protein 1 paralog (PKNH_0728800) (MSP1P). **b** Amino acid sequence identity between full-length PvMSP1P and 7 field isolates of PkMSP1P from Malaysian Borneo. **c** Amino acid sequence identity between the 19 kDa PvMSP1P and 7 field isolates of PkMSP1P from Malaysian Borneo. The highlighted-red percentages (70.2 and 86.0%) are PkMSP1P H-strain sequence identity with PvMSP1P (PVX_099975)

**Fig. 2:**
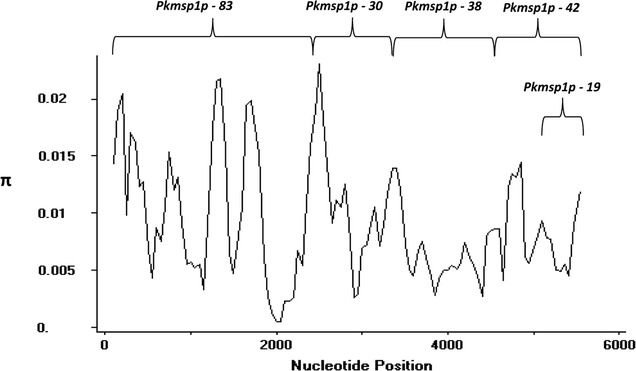
Graphical representation of nucleotide diversity (π) within 36 full-length PkMSP1P genes (n = 5622 bp) from Malaysia. The PkMSP1P domains are marked above

The Signal IP and Phobious servers detected a signal peptide in between amino acid positions 30 and 40 of the PkMSP1P protein (Additional file 3). Alignment and comparison of the amino acid sequences of the full-length *P. knowlesi* H reference strain MSP1P sequences with *P. vivax* MSP1P Sal-1 reference strain showed 70.2% identity. The schematic structure of *pkmsp1p* gene in comparison with its orthologous *pvmsp1p* is shown in Fig. 1a. The tandem repeat region (SAYSYSV)*n* and the polymorphic region (E/Q)*n* did not exist in the PkMSP1 probably due to deletion in these regions (Additional file 4). Within the full-length PkMSP1P sequences (n = 36), there were 339 polymorphic sites (6.04%) leading 164 synonymous and 175 nonsynonymous substitutions. The dimorphic nucleotides within each domain are given in the (Additional file 5). There were 174 parsimony informative sites of which 12 sites were of three variants and 165 singleton variable sites. The overall nucleotide diversity was higher (π = 0.00941 ± SD 0.00059) compared to its ortholog *P. vivax,* which is relatively conserved (Table 1) [39]. The diversity towards the N-terminal domains *pkmsp1*-*83* (π = 0.0105 ± SD 0.0007) and *30* (π = 0.0113 ± SD 0.0009) was moderately higher than the C-terminal domains *pkmsp1*-*38* (π = 0.0066 ± 0.0006), *33* (π = 0.0095 ± SD 0.0007) and *19* (π = 0.00661 ± SD 0.0007) (Table 1). The analysis with sliding window plot (window length 200 bp and step size 50 bp) also revealed that the overall diversity range from 0 to 0.02 and the C-terminal regions containing the 19 kDa domain showed lower diversity (Fig. 2). The haplotype numbers as well as the haplotype diversity of all *pkmsp1* domains were high except for the *pkmsp1p*-*19* domain (Table 1). *Pkmsp1p* genes revealed that the 12 cysteine residues within the two EGF domains at the 19 kDa domain were conserved (Fig. 1). To determine whether natural selection contributes to the polymorphism in the *pkmsp1p* full-length gene as well as at each domain, the average difference of (dN − dS) was evaluated. The significant negative value at each domain (Table 1) obtained indicated dN < dS. Thus, the full-length gene as well as all the domains appeared to be under negative or purifying selection. However, Tajima’s D, Li and Fu’s F* and D* statistics was found to be significant only for *pkmsp1p*-*38* and *19* domains (Table 1).

**Table 1:** Estimates of nucleotide diversity, natural selection, haplotype diversity and neutrality indices of *pkmsp1p*

Domain	No. samples	SNPs	No. haplotype	Diversity ± SD		dN-dS	Codon based *z* test	Taj D	Fu & Li’s D*	Fu & Li’s F*
				Haplotype	Nucleotide					
Full-length	36	339	35	0.998 ± 0.007	0.00941 ± 0.0005	− 7.43	*P* < 0.000	− 1.4	− 2.11	− 2.22
83 kDa	36	138	35	0.998 ± 0.007	0.0105 ± 0.0007	− 4.85	*P* < 0.000	− 1.09	− 1.48	− 1.59
30 kDa	36	64	34	0.997 ± 0.007	0.0113 ± 0.0009	− 3.1	*P* < 0.002	− 1.15	− 1.83	− 1.89
38 kDa	36	72	32	0.992 ± 0.009	0.0066 ± 0.0006	− 2.56	*P* < 0.01	− 1.96	− 2.94	− 3.09
								*P* < 0.05	*P* < 0.02	*P* < 0.02
33 kDa	36	49	34	0.997 ± 0.007	0.0095 ± 0.0007	− 3.56	*P* < 0.000	− 1.28	− 1.82	− 1.94
19 kDa	40	16	18	0.885 ± 0.038	0.00661 ± 0.0007	− 2.214	*P* < 0.02	− 1.75	− 2.93	− 3.02
									*P* < 0.02	*P* < 0.02

*SNPs* single nucleotide polymorphisms, *SD* standard deviation

### Phylogenetic analysis

**Fig. 3:**
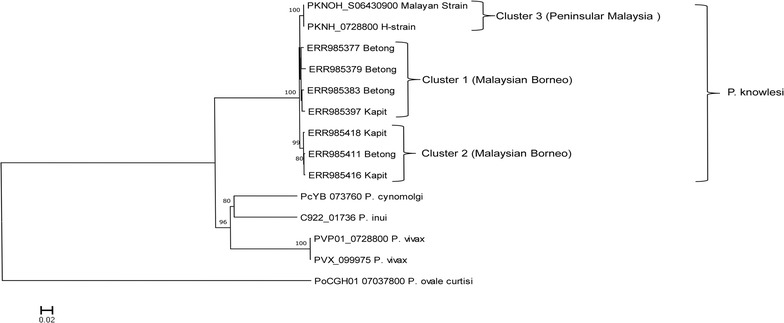
Phylogenetic relationship of MSP1P genes within ortholog *Plasmodium* species *Plasmodium vivax*, *Plasmodium knowlesi*, *Plasmodium ovale* and *Plasmodium cynomolgi* based on unrooted neighbour-joining method. The two *P. knowlesi* PkMSP1P clusters identified in Malaysian Borneo are shown as cluster 1 and cluster 2 and the two laboratory lines formed the cluster 3 from Peninsular Malaysia. Numbers at the nodes indicate bootstrap values

Phylogenetic analysis of the 9 full-length PkMSP1P amino acid sequences with other *Plasmodium* orthologs using unrooted NJ method identified two distinct *P. knowlesi* clusters from Malaysian Borneo which were supported by 100% bootstrap values (Fig. 3). The two laboratory lines the H-strain and the Malayan Strain, which originated from Peninsular Malaysia formed the third cluster (Fig. 3). These distinct-clusters were similar to the previous discovery of two distinct clusters of *P. knowlesi* parasites in clinical isolates from Sarawak, Malaysian Borneo at the genomic level [23]. The PkMSP1P was found to be more closely related to *P. cynomolgi* MSP1P compared to its ortholog in *P. vivax* and *P. ovale.*

### Genetic diversity and natural selection of PkMSP1P-19

**Fig. 4:**
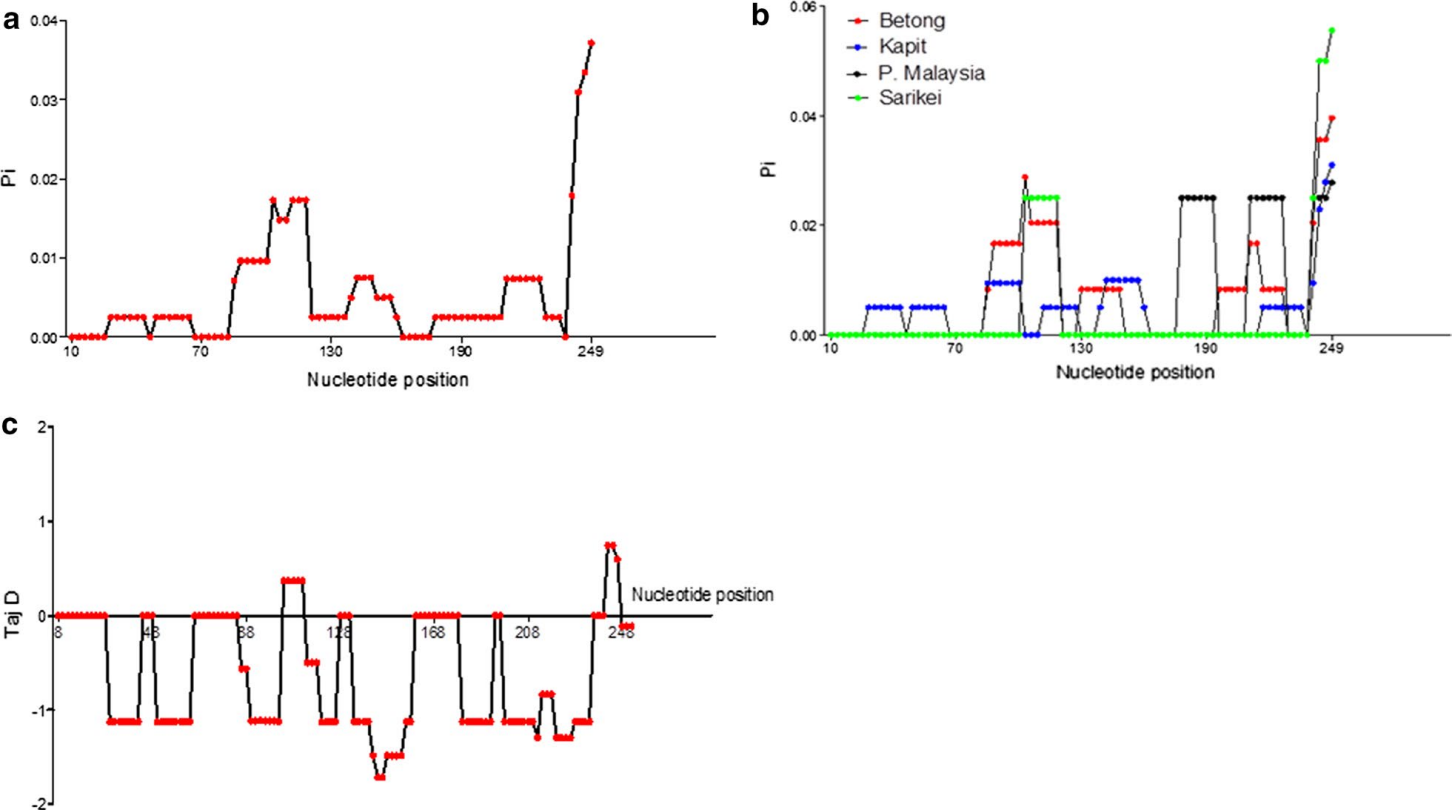
**a** Genetic diversity of PkMSP1P at the 19 kDa domain (n = 40). **b** Graphical representation of region wise diversity of PkMSP1P at the 19 kDa domain. **c** Graphical representation of Tajima’s D value at the 19 kDa domain

Amino acid sequence analysis of 40 *msp1p* genes at the PkMSP1P-19 showed that it shares 84–86% sequence identity with its ortholog PvMSP1P-19 of *P. vivax* Sal-1 (Fig. 1c). This could be explained because of the conservation of the EGF domains of MSP genes within *Plasmodium* species. There were only 16 polymorphic sites identified at the 19 kDa domain which led to 10 synonymous and 6 nonsynonymous substitutions. There were 5 parsimony informative sites and 11 singleton variable sites. Region-wise diversity using sliding window plot in DnaSP indicated that nucleotide diversity were similar in all populations (Table 2, Fig. 4b), except for the Peninsular Malaysia where higher diversity was observed within 180–200 nt position. There were 18 haplotypes identified in PkMSP1P-19 which had moderate level of haplotype diversity compared to the full-length gene (Table 1). Significant negative values for (dN − dS = − 2.214, *P* < 0.02) were observed within the domain indicating strong negative or purifying selection within the parasite population. These values were further supported by Tajima’s D and Li and Fu’s F* and D* values (Table 1). A sliding window plot of Tajima’s D value across the 19 kDa domain shows most SNPs with negative values (Fig. 4c). The amino acid polymorphism identified are shown in Additional file 6. All the 12 cysteine residues in the EGF domains were also conserved within the 40 *pkmsp1p 19* gene sequences (Additional file 7). List of 18 haplotypes identified within the 40 *pkmsp1p*-*19* gene sequences are listed in Additional file 8.

**Table 2:** Region wise diversity of *pkmsp1p*-*19*

Location	n	Diversity	SNPs	Syn.	Non-syn.
Sarikei	4	0.00581	3	2	1
Betong	12	0.00758	8	5	3
Peninsular Malaysia	4	0.00775	4	4	0
Kapit	20	0.00522	10	6	4
Overall	40	0.00661	16	10	6

*SNPs* single nucleotide polymorphisms, *Syn.* synonymous substitutions, *Non-syn.* nonsynonymous substitutions

### Haplotype network analysis of PkMSP1P- 19

**Fig. 5:**
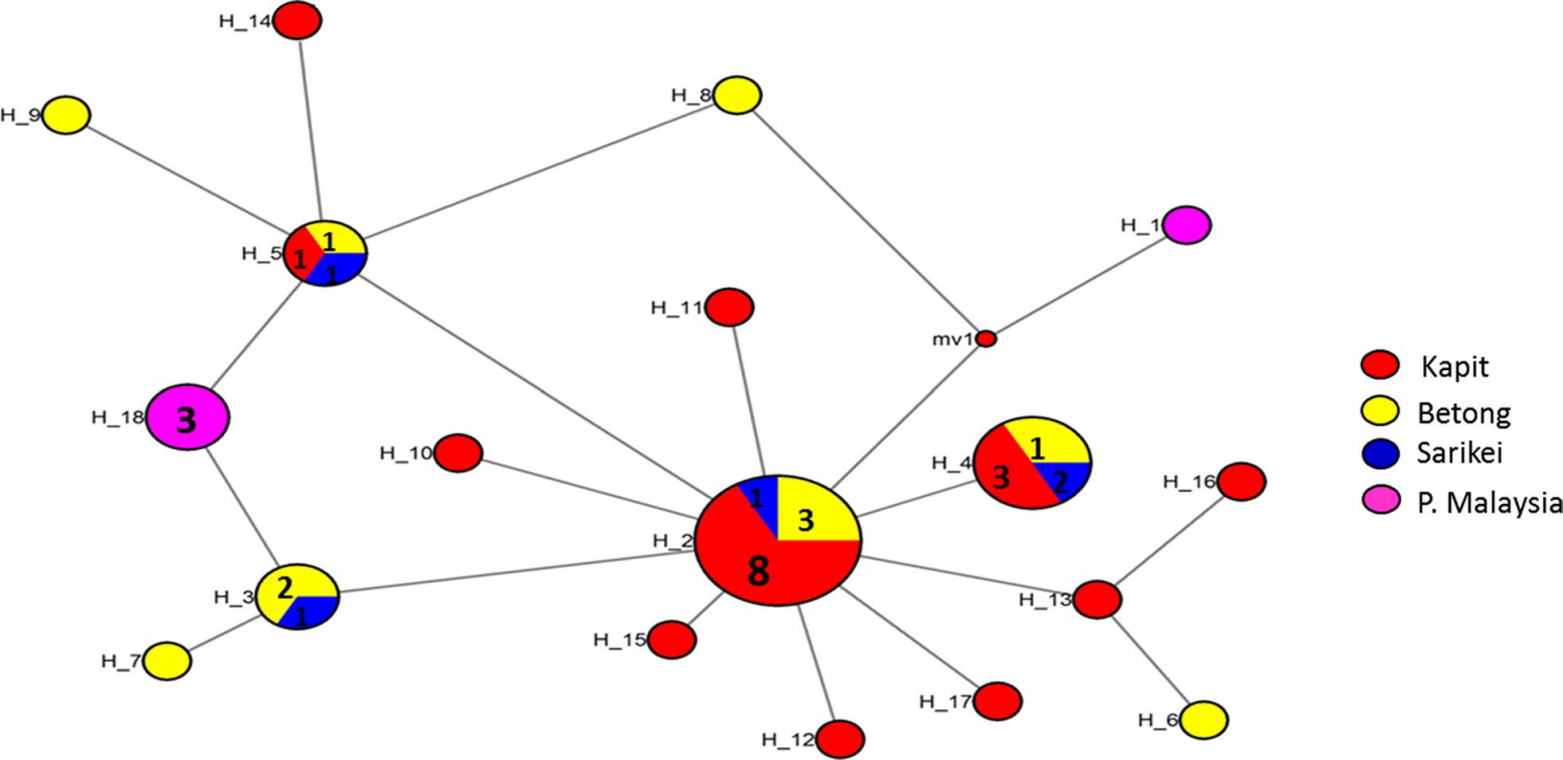
Median-joining networks of *Plasmodium knowlesi* MSP1P haplotypes from Malaysia. The genealogical haplotype network shows the relationships among the 18 haplotypes present in the 40 sequences obtained from clinical isolates from 4 geographical regions of Malaysia. Each distinct haplotype has been designated a number (H_n). Circle sizes represent the frequencies of the corresponding haplotype (the number is indicated for those that were observed > 1×). Distances between nodes are arbitrary

Intra-species nucleotide sequence variation in a phylogeographic study is more clearly observed in a haplotype network due to possibility of recombination events and the limited differences within the sequences. The network tree for PkMSP1P-19 (Fig. 5) identified 3 shared haplotype (H_2, H_4 and H_5) between Kapit, Betong and Sarikei and 1 between Betong and Sarikei (H_3). H_2 was a dominant cluster which had the highest number of samples (*n* = 12) and majority originating from Kapit (*n* = 8). Haplotypes originating from Peninsular Malaysia (H_18 and H_1) were distinct and did not cluster along with the other populations. However, limited number of samples from Peninsular Malaysia, Betong and Sarikei precludes accurate comparison in the network. Haplotypes with single isolates clustered mostly closer to the dominant haplotype H_2 (Fig. 5).

### Population genetic structure analysis based on PkMSP1P-42

Considering the shared as well as distinct *pkmsp1p* haplotypes identified, a Bayesian admixture model implemented in STRUCTURE was used to calculate the potential number of *P. knowlesi* parasite sub-populations within the four populations. K values from 1 to 6 were used for the analysis in the software to find the probable number of sub-population clusters. Significant genetic structure was found between the parasite populations when K = 4 (ΔK = 37.02) (Additional file 9 A, B), indicating 4 distinct sub-populations within the 4 geographical regions of Malaysia. Pairwise population differentiation index *F*_*ST*_ values using ARLEQUIN software also showed moderate to high genetic differentiation within the populations (Additional file 10). Very high and significant genetic differentiation was observed between parasites of Peninsular Malaysia and Kapit (*F*_*ST*_ = 0.20, *P* < 0.05) (Additional file 10). Moderate genetic differentiation was observed between populations of Sarikei and Betong (*F*_*ST*_ = 0.140) though not significant suggesting that parasitic transmission might be confined to each of the regions. However, low genetic differentiation was noted for parasites originating from Kapit and Betong which were concordant with the shared number of haplotype in the network analysis.

## Discussion

The PvMSP1P-19 is a novel vaccine candidate and has low polymorphism in field samples as well as generates protective immune response in patient serum using recombinant expressed proteins [35, 36, 39]. Thus in the present study the objective was to genetically characterize the *pkmsp1p* gene and study the level of genetic diversity, natural selection acting at the full-length PkMSP1P and 19 kDa domain. Sequence alignment of 36 full-length amino acid sequences of *pkmsp1p* genes from Malaysia showed that it shares approximately 70% sequence identity with its ortholog *pvmsp1p* and sequence identity is high (86%) towards the C-terminal 19 kDa domain. This feature is similar to the other studies on MSPs where the conservation (low levels of polymorphisms) of the EGF domains in 19 kDa domain has been reported [45]. The genetic diversity of the full-length *pkmsp1p* was high (π = 0.00941 ± SD 0.0005) compared to its ortholog *pvmsp1p* [39]. This is probably due to host immune pressure and dimorphism in *P. knowlesi* compared to its ortholog in *P. vivax*. Despite having 339 SNPs, the number synonymous and nonsynonymous substitutions was not of vast difference. This was because of high number of low frequency polymorphisms (singleton sites) at each domain leading to high haplotype diversity. The 165 singleton variable sites were detected indicated that new and rare variant were present indicating population expansion however, Li and Fu’s D* and F* did not show significant negative values. Overall, the full-length gene showed significant negative selection (− 7.43, *P* < 0.000) and similar significant negative selection has been recently reported for invasion genes *pknbpxa*, *pkmsp3*, and *pkmsp1* [27–29]. The phylogenetic tree showed separation of the *P. knowlesi* MSP1P isolates from Malaysian Borneo into 2 clusters while the laboratory lines from Peninsular Malaysia formed a third cluster. Studies on *P. knowlesi* proteins such as the DBPαII (PkDBPαII) [46], PkNBPXa [29], PkAMA1 [47] and a genomic study [23] from Borneo have also reported bifurcation of trees, indicating dimorphism of the genes at the genomic level.

The genetic diversity at PkMSP1P-19 domain was low (π = 0.0061 ± 0.00072) compared to the full-length gene as well as the rest of the N terminal domains indicating conservation of the two EGF domains as reported in its ortholog PvMSP1P [39]. Interestingly, the 12 cysteine residues within the two EGF domains were conserved in all *P. knowlesi* isolates used in this study. Both PkMSP1P and PvMSP1P showed similar conservation of the cysteine residues indicating they might share similar protein structure and function. This is a significant finding as the binding site of PvMSP1P and PkMSP1P are within these EGF domains and antigenicity has been observed in both *P. vivax* and *P. knowlesi* patient serum samples (Muh et al., unpublished data). Significant negative/purifying selection was observed within the 19 kDa domain indicating functional constraints within the parasite population.

Haplotype network analysis identified 3 predominantly shared *pkmsp1p* haplotypes (between Kapit, Betong and Sarikei) but no shared haplotypes with P. Malaysia. This finding is significant as similar geographical sub-population cluster was noticed for recent *P. knowlesi* mitochondrial *cox* 1, *ssrRNA* and *csp* genetic study in Malaysia [24, 48]. Interestingly, in this study some unique haplotypes (H_1, from Peninsular Malaysia) which did not cluster within the predominant haplotypes (Fig. 5) were also noted. H_1 and H_8 from Peninsular Malaysia were distantly apart probably because these laboratory lines were isolated from distant geographical locations. Additional population structure analyses showed moderate genetic differentiation between parasite populations originating from Sarikei and Betong (Fst = 0.14, *P* > 0.05) and very high between P. Malaysia and Kapit (Fst = 0.20, *P* < 0.05) (Additional file 10). Results were significant with robust Bayesian structure analysis where 4 different sub-populations were identified however, higher sample number are required from Sarikei and Peninsular Malaysia for accurate determination of the population structure. A recent genomic study identified host-specific sub-populations of *P. knowlesi* infections and has indicated a recombination event in the sexual stages of the parasite [49]. These observations might indicate that humans are susceptible to infection by any of the *P. knowlesi* populations circulating in these regions.

## Conclusion

The present study is the first to investigate genetic diversity, natural selection and population structure of the *pkmsp1p* gene. High level of genetic diversity was observed in the full-length PkMSP1P gene and the C-terminal 19 kDa region appeared to be relatively conserved and under strong purifying selection. Shared haplotypes were observed for the 19 kDa domain. Future studies should investigate the diversity of PkMSP1P 19 kDa domain among *P. knowlesi* isolates from all over Malaysia.

## Additional files

**Additional file 1: Figure S1.** Geographical origin of samples used in this study.

**Additional file 2: Table S1.** Accession number of PkMSP1P sequences used in the study and their geographical origin.

**Additional file 3: Figure S2.** Signal peptide prediction by (A) Signal IP server and (B) Phobious server. Signal peptide was predicted in between amino acid positions 30 to 40.

**Additional file 4: Figure S3.** Alignment showing the deletion of the (A) tandem repeat regions and the (B) polymorphic regions in PkMSP1P in comparison to its ortholog PvMSP1P.

**Additional file 5.** Nucleotide polymorphism and dimorphism within 7 full-length PkMSP1P sequences from Malaysian Borneo. Dimorphic bases in each domain is boxed.

**Additional file 6.** Amino acid polymorphism within 40 PkMSP1P sequences from Malaysia.

**Additional file 7.** Amino acid alignment of PvMS1P and PkMSP1P 19 kDa domain. Conserved regions are highlighted in red above along with the 12 conserved cysteine residues (marked as asterisk below).

**Additional file 8.** List of the 18 haplotypes identified within the *pkmsp1p-19*.

**Additional file 9.** (A) K= 4, Population structure of *Plasmodium knowlesi* in Malaysia based on MSP1P. (B) A peak for Δ*K* (37.02) at *K* = 4 suggests that 4 populations best fit the data.

**Additional file 10.** Population differentiation values (*F*_*ST*_) based on *pkmsp1p-42*.

## Abbreviations

MSP1P: merozoite surface protein 1 paralog
kDa: kilodalton

## References

[1] WHO (2016). World malaria report.

[2] White NJ (2008). *Plasmodium knowlesi*: the fifth human malaria parasite. Clin Infect Dis.

[3] Cox-Singh J, Davis TM, Lee KS, Shamsul SS, Matusop A, Ratnam S (2008). *Plasmodium knowlesi* malaria in humans is widely distributed and potentially life threatening. Clin Infect Dis.

[4] Singh B, Kim Sung L, Matusop A, Radhakrishnan A, Shamsul SS, Cox-Singh J (2004). A large focus of naturally acquired *Plasmodium knowlesi* infections in human beings. Lancet.

[5] Garnham PCC (1966). Malaria parasites and other haemosporidia.

[6] Ahmed MA, Cox-Singh J (2015). *Plasmodium knowlesi*—an emerging pathogen. ISBT Sci Ser.

[7] Vythilingam I, Noorazian YM, Huat TC, Jiram AI, Yusri YM, Azahari AH (2008). *Plasmodium knowlesi* in humans, macaques and mosquitoes in peninsular Malaysia. Parasit Vectors.

[8] Barber BE, William T, Jikal M, Jilip J, Dhararaj P, Menon J (2011). *Plasmodium knowlesi* malaria in children. Emerg Infect Dis.

[9] Ng OT, Ooi EE, Lee CC, Lee PJ, Ng LC, Pei SW (2008). Naturally acquired human *Plasmodium knowlesi* infection, Singapore. Emerg Infect Dis.

[10] Jiang N, Chang Q, Sun X, Lu H, Yin J, Zhang Z (2010). Co-infections with *Plasmodium knowlesi* and other malaria parasites, Myanmar. Emerg Infect Dis.

[11] Van den Eede P, Van HN, Van Overmeir C, Vythilingam I, Duc TN, le Hung X (2009). Human *Plasmodium knowlesi* infections in young children in central Vietnam. Malar J.

[12] Figtree M, Lee R, Bain L, Kennedy T, Mackertich S, Urban M (2010). *Plasmodium knowlesi* in human, Indonesian Borneo. Emerg Infect Dis.

[13] Luchavez J, Espino F, Curameng P, Espina R, Bell D, Chiodini P (2008). Human infections with *Plasmodium knowlesi*, the Philippines. Emerg Infect Dis.

[14] Khim N, Siv S, Kim S, Mueller T, Fleischmann E, Singh B (2011). *Plasmodium knowlesi* infection in humans, Cambodia, 2007–2010. Emerg Infect Dis.

[15] Tyagi RK, Das MK, Singh SS, Sharma YD (2013). Discordance in drug resistance-associated mutation patterns in marker genes of *Plasmodium falciparum* and *Plasmodium knowlesi* during coinfections. J Antimicrob Chemother.

[16] Sermwittayawong N, Singh B, Nishibuchi M, Sawangjaroen N, Vuddhakul V (2012). Human *Plasmodium knowlesi* infection in Ranong province, southwestern border of Thailand. Malar J.

[17] Yusof R, Lau YL, Mahmud R, Fong MY, Jelip J, Ngian HU (2014). High proportion of knowlesi malaria in recent malaria cases in Malaysia. Malar J.

[18] Daneshvar C, Davis TM, Cox-Singh J, Rafa’ee MZ, Zakaria SK, Divis PC (2009). Clinical and laboratory features of human *Plasmodium knowlesi* infection. Clin Infect Dis.

[19] William T, Menon J, Rajahram G, Chan L, Ma G, Donaldson S (2011). Severe *Plasmodium knowlesi* malaria in a tertiary care hospital, Sabah, Malaysia. Emerg Infect Dis.

[20] Willmann M, Ahmed A, Siner A, Wong IT, Woon LC, Singh B (2012). Laboratory markers of disease severity in *Plasmodium knowlesi* infection: a case control study. Malar J.

[21] Pinheiro MM, Ahmed MA, Millar SB, Sanderson T, Otto TD, Lu WC (2015). *Plasmodium knowlesi* genome sequences from clinical isolates reveal extensive genomic dimorphism. PLoS ONE.

[22] Divis PC, Singh B, Anderios F, Hisam S, Matusop A, Kocken CH (2015). Admixture in humans of twodivergent *Plasmodium knowlesi* populations associated with different macaque host species. PLoS Pathog.

[23] Assefa S, Lim C, Preston MD, Duffy CW, Nair MB, Adroub SA (2015). Population genomic structure and adaptation in the zoonotic malaria parasite *Plasmodium knowlesi*. Proc Natl Acad Sci USA.

[24] Yusof R, Ahmed MA, Jelip J, Ngian HU, Mustakim S, Hussin HM (2016). Phylogeographic evidence for 2 genetically distinct zoonotic *Plasmodium knowlesi* Parasites, Malaysia. Emerg Infect Dis.

[25] Gosling R, von Seidlein L (2016). The future of the RTS, S/AS01 malaria vaccine: an alternative development plan. PLoS Med..

[26] Ahmed AM, Pinheiro MM, Divis PC, Siner A, Zainudin R, Wong IT (2014). Disease progression in *Plasmodium knowlesi* malaria is linked to variation in invasion gene family members. PLoS Negl Trop Dis.

[27] Yap NJ, Goh XT, Koehler AV, William T, Yeo TW, Vythilingam I (2017). Genetic diversity in the C-terminus of merozoite surface protein 1 among *Plasmodium knowlesi* isolates from Selangor and Sabah Borneo, Malaysia. Infect Genet Evol.

[28] De Silva JR, Lau YL, Fong MY (2017). Genetic clustering and polymorphism of the merozoite surface protein-3 of *Plasmodium knowlesi* clinical isolates from Peninsular Malaysia. Parasit Vectors.

[29] Ahmed MA, Fong MY, Lau YL, Yusof R (2016). Clustering and genetic differentiation of the normocyte binding protein (nbpxa) of *Plasmodium knowlesi* clinical isolates from Peninsular Malaysia and Malaysia Borneo. Malar J.

[30] Waters AP, Higgins DG, McCutchan TF (1993). Evolutionary relatedness of some primate models of *Plasmodium*. Mol Biol Evol.

[31] Perera KL, Handunnetti SM, Holm I, Longacre S, Mendis K (1998). Baculovirus merozoite surface protein 1 C-terminal recombinant antigens are highly protective in a natural primate model for human *Plasmodium vivax* malaria. Infect Immun.

[32] Valderrama-Aguirre A, Quintero G, Gomez A, Castellanos A, Perez Y, Mendez F (2005). Antigenicity, immunogenicity, and protective efficacy of *Plasmodium vivax* MSP1 PV200l: a potential malaria vaccine subunit. Am J Trop Med Hyg.

[33] Marshall VM, Tieqiao W, Coppel RL (1998). Close linkage of three merozoite surface protein genes on chromosome 2 of *Plasmodium falciparum*. Mol Biochem Parasitol.

[34] Black CG, Wang L, Wu T, Coppel RL (2003). Apical location of a novel EGF-like domain-containing protein of *Plasmodium falciparum*. Mol Biochem Parasitol.

[35] Cheng Y, Wang Y, Ito D, Kong DH, Ha KS, Chen JH (2013). The *Plasmodium vivax* merozoite surface protein 1 paralog is a novel erythrocyte-binding ligand of *P. vivax*. Infect Immun.

[36] Cheng Y, Shin EH, Lu F, Wang B, Choe J, Tsuboi T (2014). Antigenicity studies in humans and immunogenicity studies in mice: an MSP1P subdomain as a candidate for malaria vaccine development. Microbes Infect.

[37] Changrob S, Leepiyasakulchai C, Tsuboi T, Cheng Y, Lim CS, Chootong P (2015). Naturally-acquired cellular immune response against *Plasmodium vivax* merozoite surface protein-1 paralog antigen. Malar J.

[38] O’Donnell RA, Saul A, Cowman AF, Crabb BS (2000). Functional conservation of the malaria vaccine antigen MSP-119across distantly related *Plasmodium* species. Nat Med.

[39] Wang Y, Kaneko O, Sattabongkot J, Chen JH, Lu F, Chai JY (2011). Genetic polymorphism of *Plasmodium vivax* msp1p, a paralog of merozoite surface protein 1, from worldwide isolates. Am J Trop Med Hyg.

[40] Petersen TN, Brunak S, von Heijne G, Nielsen H (2011). SignalP 4.0: discriminating signal peptides from transmembrane regions. Nat Methods.

[41] Kall L, Krogh A, Sonnhammer EL (2007). Advantages of combined transmembrane topology and signal peptide prediction—the Phobius web server. Nucleic Acids Res.

[42] Librado P, Rozas J (2009). DnaSP v5: a software for comprehensive analysis of DNA polymorphism data. Bioinformatics.

[43] Tajima F (1989). Statistical method for testing the neutral mutation hypothesis by DNA polymorphism. Genetics.

[44] Tamura K, Peterson D, Peterson N, Stecher G, Nei M, Kumar S (2011). MEGA5: molecular evolutionary genetics analysis using maximum likelihood, evolutionary distance, and maximum parsimony methods. Mol Biol Evol.

[45] Chaurio RA, Pacheco MA, Cornejo OE, Durrego E, Stanley CE, Castillo AI (2016). Evolution of the transmission-blocking vaccine candidates Pvs28 and Pvs25 in *Plasmodium vivax*: geographic differentiation and evidence of positive selection. PLoS Negl Trop Dis.

[46] Fong MY, Lau YL, Chang PY, Anthony CN (2014). Genetic diversity, haplotypes and allele groups of Duffy binding protein (PkDBPalphaII) of *Plasmodium knowlesi* clinical isolates from Peninsular Malaysia. Parasit Vectors.

[47] Faber BW, Abdul Kadir K, Rodriguez-Garcia R, Remarque EJ, Saul FA, Vulliez-Le Normand B (2015). Low levels of polymorphisms and no evidence for diversifying selection on the *Plasmodium knowlesi* Apical Membrane Antigen 1 gene. PLoS ONE.

[48] Fong MY, Ahmed MA, Wong SS, Lau YL, Sitam F (2015). Genetic diversity and natural selection of the *Plasmodium knowlesi* circumsporozoite protein nonrepeat regions. PLoS ONE.

[49] Diez Benavente E, Florez de Sessions P, Moon RW, Holder AA, Blackman MJ, Roper C (2017). Analysis of nuclear and organellar genomes of *Plasmodium knowlesi* in humans reveals ancient population structure and recent recombination among host-specific subpopulations. PLoS Genet.

