# Supplementary figures and images for: Genetic diversity and natural selection of Plasmodium knowlesi merozoite surface protein 1 paralog gene in Malaysia
Source: Malar J. 2018 Mar 14;17:115. doi: 10.1186/s12936-018-2256-y (PMC5853062; doi:10.1186/s12936-018-2256-y)

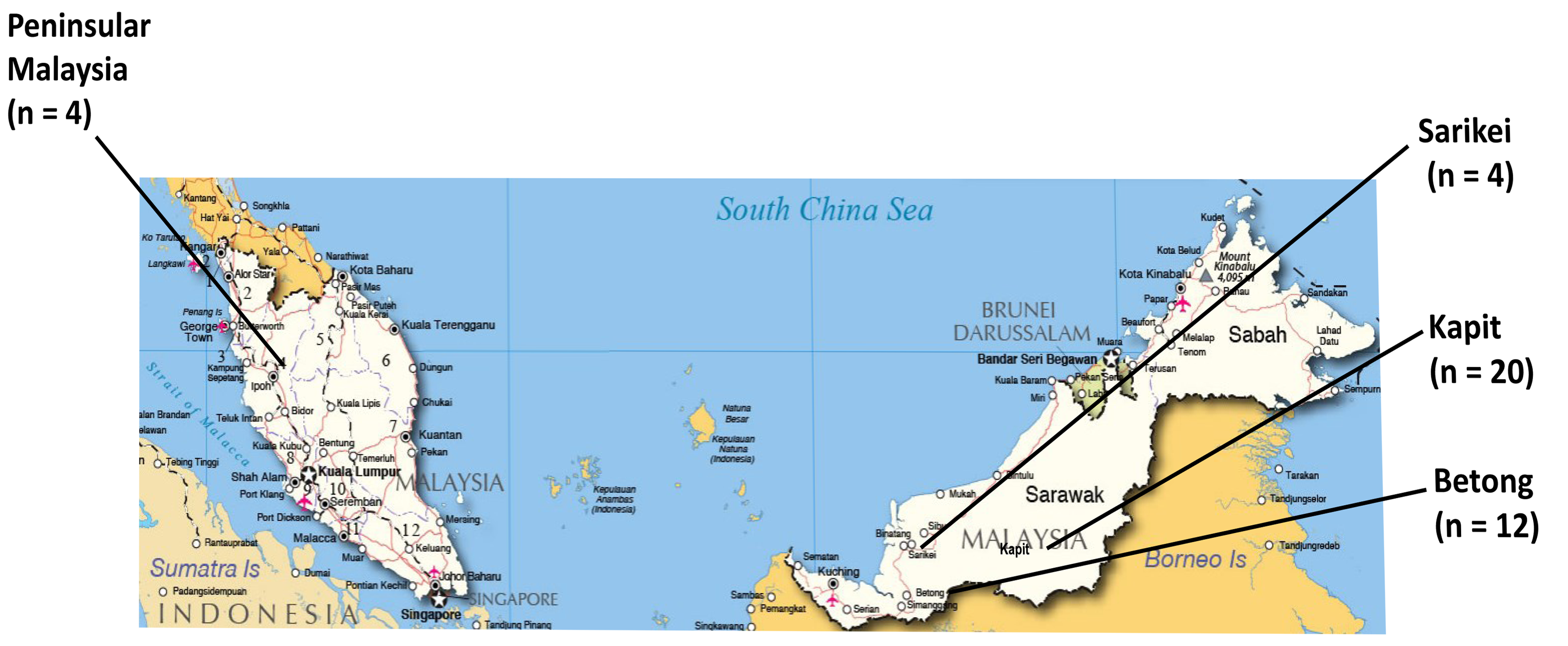

Supplement: Supplementary file 1 — Additional file 1: Figure S1. Geographical origin of samples used in this study. [file 12936_2018_2256_MOESM1_ESM.tif]

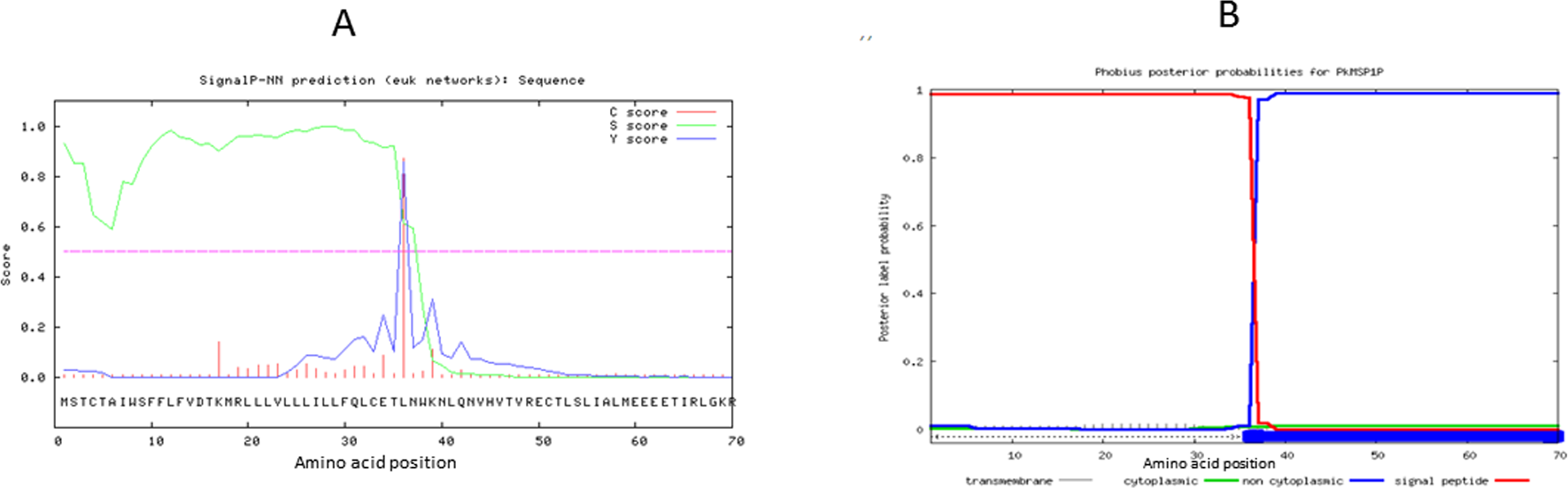

Supplement: Supplementary file 3 — Additional file 3: Figure S2. Signal peptide prediction by (A) Signal IP server and (B) Phobious server. Signal peptide was predicted in between amino acid positions 30 to 40. [file 12936_2018_2256_MOESM3_ESM.tif]

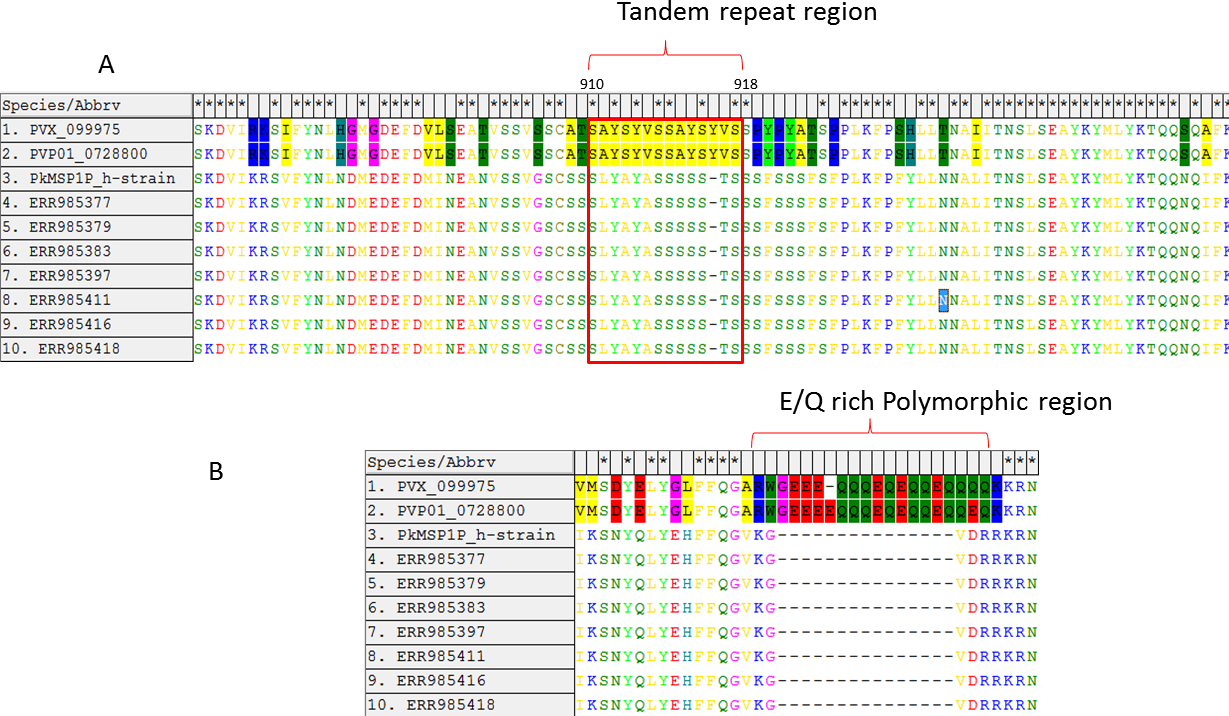

Supplement: Supplementary file 4 — Additional file 4: Figure S3. Alignment showing the deletion of the (A) tandem repeat regions and the (B) polymorphic regions in PkMSP1P in comparison to its ortholog PvMSP1P. [file 12936_2018_2256_MOESM4_ESM.tif]

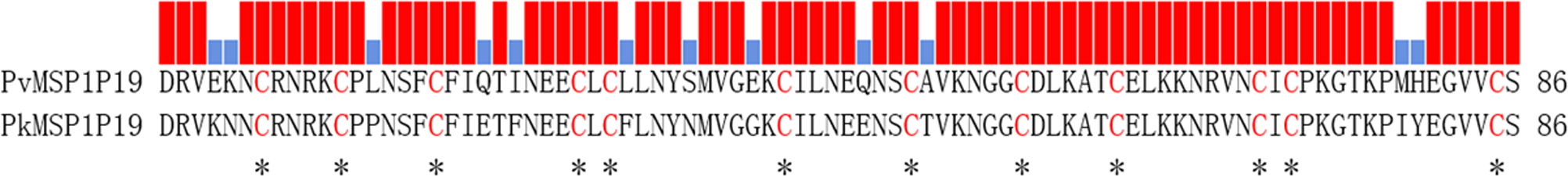

Supplement: Supplementary file 7 — Additional file 7. Amino acid alignment of PvMS1P and PkMSP1P 19 kDa domain. Conserved regions are highlighted in red above along with the 12 conserved cysteine residues (marked as asterisk below). [file 12936_2018_2256_MOESM7_ESM.tif]

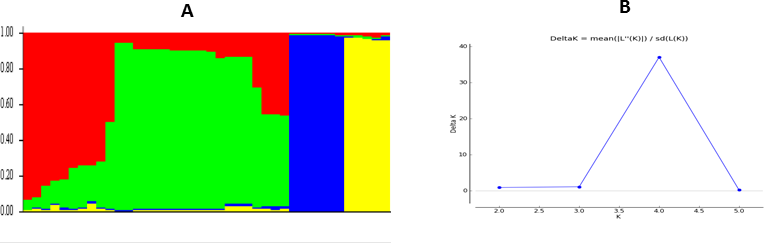

Supplement: Supplementary file 9 — Additional file 9. (A) K= 4, Population structure of Plasmodium knowlesi in Malaysia based on MSP1P. (B) A peak for ΔK (37.02) at K = 4 suggests that 4 populations best fit the data. [file 12936_2018_2256_MOESM9_ESM.tif]
