# Supplementary material for: Genetic diversity and natural selection of Plasmodium knowlesi merozoite surface protein 1 paralog gene in Malaysia
Source: Malar J. 2018 Mar 14;17:115. doi: 10.1186/s12936-018-2256-y (PMC5853062; doi:10.1186/s12936-018-2256-y)
Supplement: Supplementary file 2 — Additional file 2: Table S1. Accession number of PkMSP1P sequences used in the study and their geographical origin. [file 12936_2018_2256_MOESM2_ESM.docx]

**Additional file 2 Table S1. Study samples and origin**

| **No.** | **Sample** | **Area** | **Year** |
| --- | --- | --- | --- |
| 1 | **ERR274221** | Sarikei | 2012/3 |
| 2 | **ERR274222** | Sarikei | 2012/3 |
| 3 | **ERR366425** | Sarikei | 2012/3 |
| 4 | **ERR366426** | Sarikei | 2012/3 |
| 5 | **ERR985374** | Betong | 2012/3 |
| 6 | **ERR985376** | Betong | 2012/3 |
| 7 | **ERR985377** | Betong | 2012/3 |
| 8 | **ERR985378** | Betong | 2012/3 |
| 9 | **ERR985379** | Betong | 2012/3 |
| 10 | **ERR985380** | Betong | 2012/3 |
| 11 | **ERR985381** | Betong | 2012/3 |
| 12 | **ERR985382** | Betong | 2012/3 |
| 13 | **ERR985383** | Betong | 2012/3 |
| 14 | **ERR985384** | Betong | 2012/3 |
| 15 | **ERR985385** | Kapit | 2012/3 |
| 16 | **ERR985386** | Kapit | 2012/3 |
| 17 | **ERR985387** | Kapit | 2012/3 |
| 18 | **ERR985388** | Kapit | 2012/3 |
| 19 | **ERR985390** | Kapit | 2012/3 |
| 20 | **ERR985392** | Kapit | 2012/3 |
| 21 | **ERR985393** | Kapit | 2012/3 |
| 22 | **ERR985394** | Kapit | 2012/3 |
| 23 | **ERR985395** | Kapit | 2012/3 |
| 24 | **ERR985396** | Kapit | 2012/3 |
| 25 | **ERR985397** | Kapit | 2012/3 |
| 26 | **ERR985404** | Kapit | 2012/3 |
| 27 | **ERR985405** | Kapit | 2012/3 |
| 28 | **ERR985406** | Kapit | 2012/3 |
| 29 | **ERR985407** | Kapit | 2012/3 |
| 30 | **ERR985408** | Kapit | 2012/3 |
| 31 | **ERR985409** | Kapit | 2012/3 |
| 32 | ERR985410 | Betong | 2012/3 |
| 33 | **ERR985411** | Betong | 2012/3 |
| 34 | **ERR985416** | Kapit | 2012/3 |
| 35 | ERR985417 | Kapit | 2012/3 |
| 36 | **ERR985418** | Kapit | 2012/3 |
| 37 | SRR2222335 | P. Malaysia |  |
| 38 | SRR3135172 | P. Malaysia |  |
| 39 | **Malayan Strain Pk1A PKNOH_S06430900** | P. Malaysia |  |
| 40 | **H-strain(PKNH_0728800)** | P. Malaysia |  |

P: Peninsular

Isolates with bold accession numbers were used for full-length characterisation of *pkmsp1p* gene.

P: Peninsular
