# Supplementary material for: Genetic diversity and natural selection of Plasmodium knowlesi merozoite surface protein 1 paralog gene in Malaysia
Source: Malar J. 2018 Mar 14;17:115. doi: 10.1186/s12936-018-2256-y (PMC5853062; doi:10.1186/s12936-018-2256-y)
Supplement: Supplementary file 6 — Additional file 6. Amino acid polymorphism within 40 PkMSP1P sequences from Malaysia. [file 12936_2018_2256_MOESM6_ESM.docx]

**Amino acid polymorphism within 40 PkMSP1P sequences from Malaysia**
