# Supplementary material for: Genetic diversity and natural selection of Plasmodium knowlesi merozoite surface protein 1 paralog gene in Malaysia
Source: Malar J. 2018 Mar 14;17:115. doi: 10.1186/s12936-018-2256-y (PMC5853062; doi:10.1186/s12936-018-2256-y)
Supplement: Supplementary file 8 — Additional file 8. List of the 18 haplotypes identified within the pkmsp1p-19. [file 12936_2018_2256_MOESM8_ESM.docx]

Hap_1 GCCTGGAAGTGTGGGG

Hap_2 GCCTGGAAGCGCGGGG

Hap_3 GCCTTGAAGCGCGGGG

Hap_4 GCCTGGAAGCGCGGAG

Hap_5 GCCTGGAAGCGCGAGG

Hap_6 GCTTGGAAGCACGGGG

Hap_7 GCCTTGGAGCGCGGGG

Hap_8 GCCTGGAAGCGTGAGG

Hap_9 GCCCGGAAGCGCGAGG

Hap_10 GCCTGGAAGCGCGGGA

Hap_11 GCCTGAAAGCGCGGGG

Hap_12 GCCTGGAGGCGCGGGG

Hap_13 GCTTGGAAGCGCGGGG

Hap_14 GCCTGGAAGCGCAAGG

Hap_15 ACCTGGAAGCGCGGGG

Hap_16 GCTTGGAAACGCGGGG

Hap_17 GTCTGGAAGCGCGGGG

Hap_18 GCCTTGAAGCGCGAGG
