# Supplementary material for: Genetic diversity and natural selection of Plasmodium knowlesi merozoite surface protein 1 paralog gene in Malaysia
Source: Malar J. 2018 Mar 14;17:115. doi: 10.1186/s12936-018-2256-y (PMC5853062; doi:10.1186/s12936-018-2256-y)
Supplement: Supplementary file 10 — Additional file 10. Population differentiation values (FST) based on pkmsp1p-42. [file 12936_2018_2256_MOESM10_ESM.docx]

**Additional file 10: Population differentiation values (*F_ST_*) from each region of Malaysia**

| Location | *F_ST_* values* | | | |
| --- | --- | --- | --- | --- |
|  | Peninsular Malaysia | Sarikei | Kapit | Betong |
| Peninsular Malaysia | - | - | - | - |
| Sarikei | 0.087 | - | - | - |
| Kapit | 0.200** | 0.052 | - | - |
| Betong | 0.071 | 0.140 | 0.017 | - |

** P <0.05
