# Supplementary material for: Genetic diversity and natural selection of Plasmodium knowlesi merozoite surface protein 1 paralog gene in Malaysia
Source: Malar J. 2018 Mar 14;17:115. doi: 10.1186/s12936-018-2256-y (PMC5853062; doi:10.1186/s12936-018-2256-y)
Supplement: Supplementary file 6 [file 12936_2018_2256_MOESM5_ESM.docx]

Additional file 5

	Nucleotide Positions						
	SP	83 kDa	30 kDa	38 kDa	33 kDa	19 kDa	GPI
Acession Numbers		111111111111111111111111111111222	222222222222222222333333	3333333333333333334444444444	44444444444444444555555555	5555	55
		111122333344556667777788888899001222223333333455666777777788133	334444455555577778000222	2233334444466677990112222334	55566777777788999000111112	5555	55
	89	233316578801471585778800033901574567790033567188279022445812722	342377700148805688667123	8905680039926903997094579672	44836556677933357248022592	1445	66
	26	135664430810635272142928967095677100332408196127643745239537802	477902535266791613368780	2467761385891750235355059726	58152264505716715896015706	1155	24
ERR985377	CG	CCTGTGGAGGACCCGATAAAGGAACAGCTTTGGTTACCCGGGAAGACTCCGCAGGAAAAACAC	TCCTGGCCACCAAAGGCGATGGCG	GCATCAACAATAACCGGCACTGCATGGA	ACGGTGCCGCGCGCGCATGCGCGCTT	CGGT	TT
ERR985379	A.	.....C.....A.A...CGG....AG...A..CG.G......CG.C..T..............	.T.....T.A......T.....A.	..G.T........T.CCT..C.....A.	G...CA.G..A.......C.......	TA.C	GC
ERR985383	.A	.ACA.C..A....A...CGGAA.......A......G..TCA.CG....TTA........G...	..TC..........T.........	..GCT......G...C.....A......	.G..CA.G.AAG.....C........	....	..
ERR985397	..	...T.C...........CGG........CA.T...G..TCATC.......A.......T....	..........T.............	..GCT......GG.AC..GA........	.G..CA.GC.A..T...C..A.....	....	..
ERR985411	..	T.CAC.........T.CCGG..GT..CT.AC..GGGTAT...CGT.TC...TGCCTGT..AGT	C.TCTAT.G..GGG.T.AGAAA.T	TTG.TCCTGGAG...C......AGC..G	GGATCA.G..A...T.GC.G..CTCC	..A.	..
ERR985416	..	T.CAC.A..AG...TCCCGG..GT..C..AC..GGGTAT...CGT.TC...TGCCTGT..AG.	C.TCTAT.G..GGG.T.AGA.A..	TTG.TCCTGGAG...C......AGCA.G	GGATCA.G..A...T.GC.G..C.CC	..A.	..
ERR985418	.A	T.CAC.AG....A.TCCCGG..GT..C..AC..GGGTAT...CGT.TC...TG.CTGT..AG.	C.TCTAT.G..GGG.T.AGA.A..	TTG.TCCTGGAG...C......AGC..G	GGATC.TG..A.T..TGC.G.AC.CC	..A.	..
